# Divergent prognostic effects of pre-existing and treatment-emergent thyroid dysfunction in patients treated with immune checkpoint inhibitors

**DOI:** 10.1007/s00262-022-03151-2

**Published:** 2022-01-24

**Authors:** Mitchell S. von Itzstein, Amrit S. Gonugunta, Yiqing Wang, Thomas Sheffield, Rong Lu, Sadia Ali, Farjana J. Fattah, Donglu Xie, Jennifer Cai, Yang Xie, David E. Gerber

**Affiliations:** 1 Department of Internal Medicine, Division of Hematology and Oncology, Harold C. Simmons Comprehensive Cancer Center, University of Texas, Southwestern Medical Center, 5323 Harry Hines Blvd., Mail Code 8852, Dallas, TX 75390-8852 USA; 2 Harold C. Simmons Comprehensive Cancer Center, University of Texas, Southwestern Medical Center, Dallas, TX 75390 USA; 3 School of Medicine, University of Texas, Southwestern Medical Center, Dallas, TX 75390 USA; 4 Department of Population and Data Sciences, University of Texas, Southwestern Medical Center, Dallas, TX 75390 USA; 5 Department of Internal Medicine, Division of Endocrinology, University of Texas, Southwestern Medical Center, Dallas, TX 75390 USA; 6 Academic Information Systems, University of Texas, Southwestern Medical Center, Dallas, TX 75390 USA; 7 Present Address: Stanford University, Palo Alto, CA USA

**Keywords:** Autoimmune, Endocrine, Immune-related adverse event, Immunotherapy, Thyroid dysfunction, Thyroid stimulating hormone (TSH)

## Abstract

**Background:**

Thyroid dysfunction is among the most common autoimmune diseases and immune checkpoint inhibitor (ICI)-induced immune-related adverse events (irAE). We determined the association between longitudinal thyroid function and clinical outcomes in patients treated with ICI.

**Methods:**

We identified all patients treated with ICI at UT Southwestern Medical Center from January 1, 2011, through December 31, 2020. We defined normal thyroid stimulating hormone (TSH) and free thyroxine (FT4) levels according to institutional reference range. We defined clinical thyroid dysfunction using established criteria incorporating labs and treatment. We determined the association between thyroid function and overall survival (OS) using Kaplan–Meier curves, log-rank tests, and multivariate Cox proportional hazards model.

**Results:**

A total of 1781 patients were included in analyses, of whom 381 (21%) had abnormal baseline TSH. Patients with abnormal baseline TSH were more likely to be female, have kidney cancer, and initiate levothyroxine after ICI initiation (all *P* < 0.001). Patients with abnormal baseline TSH had inferior OS (median 16 vs 27 months; *P* < 0.001). Among patients with normal baseline TSH, those who had abnormal TSH after ICI initiation had improved OS (median 41 vs 22 months; *P* < 0.001). In a multivariate Cox model, abnormal baseline TSH was associated with worse OS (HR 1.62; 95% CI, 1.30–2.02; *P* < 0.001), while initiation of levothyroxine after ICI initiation was associated with improved OS (HR 0.62; 95% CI, 0.44–0.88; *P* = 0.008).

**Conclusions:**

ICI-induced thyroid dysfunction is associated with improved survival, although abnormal TSH prior to ICI initiation is associated with inferior survival.

**Precis:**

Thyroid abnormalities occur commonly in the general population and as immunotherapy toxicities. We found that immunotherapy-induced thyroid dysfunction is associated with better survival, but pre-existing thyroid abnormalities convey worse outcomes.

**Supplementary Information:**

The online version contains supplementary material available at 10.1007/s00262-022-03151-2.

## Introduction

Thyroid function abnormalities represent one of the most common immune-related adverse events (irAE) seen with immune checkpoint inhibitors (ICI), occurring in 10–40% of patients [1–3]. Because these toxicities are largely unpredictable, may occur over a wide time-range [4–7], and may present with non-specific symptoms [8–10], patients receiving ICI generally undergo regular monitoring of thyroid function, including assessment prior to ICI initiation. Aside from monitoring of certain tyrosine kinase inhibitors (TKIs) typically used to treat kidney cancer, assessment of thyroid function is generally not performed for patients treated with conventional cytotoxic chemotherapy or with molecularly targeted therapies. Therefore, immunotherapy provides a unique opportunity to examine thyroid function in diverse oncology populations.

For patients undergoing ICI for cancer therapy, the approach to pre-existing thyroid dysfunction is unique among comorbidities. Autoimmune disease (Hashimoto’s thyroiditis) accounts for the majority of hypothyroidism cases [11, 12], a condition that affects up to 5% of the general population [12, 13]. However, in contrast to other autoimmune diseases, because this clinical event is transient (albeit often causing lifelong organ dysfunction) and not treated with chronic immunosuppression, thyroid dysfunction is not considered when determining eligibility for ICI. Similarly, ICI-induced thyroid dysfunction usually occurs as a transient thyroiditis event that progresses to potentially lifelong hypothyroidism and is generally treated with thyroid hormone replacement for hypothyroidism or beta blockers with or without inhibitors of thyroid hormone production for hyperthyroidism [14]. Steroids and other immune modulators are rarely used.

As observed with other irAE [15–17], the occurrence of ICI-induced thyroid dysfunction appears associated with improved clinical outcomes [18, 19], an observation that may reflect cross-reactivity between anti-tumor and autoimmune responses or even germline genetic factors [20, 21]. Given the frequency of pre-existing and ICI-induced thyroid disease, we performed a longitudinal analysis of thyroid function tests, symptoms, treatment, and outcomes in a population receiving ICI. To account for potential confounding by other treatments affecting thyroid function, we also recorded receipt of TKIs with known potential thyroid toxicity.

## Methods

### Data source, study population and data collection

This study was approved by the UT Southwestern Institutional Review Board (IRB #STU 082015-053). Using the clinical research data warehouse at UT Southwestern Medical Center, we identified patients with cancer (age ≥ 18 years) who received ICI between January 1, 2011, and December 31, 2020. The following ICI were used as search terms: atezolizumab, avelumab, cemiplimab, durvalumab, ipilimumab, nivolumab and pembrolizumab. For each case, we identified the following demographic and disease-related variables: age, sex, race, ethnicity, cancer type, and ICI received. We collected thyroid function tests [thyroid stimulating hormone (TSH) and free thyroxine (FT4)], receipt of thyroid replacement (levothyroxine), any treatment of hyperthyroidism [methimazole, propylthiouracil (PTU), radioiodine ablation, surgery], and variables that could confound interpretation of thyroid tests [amiodarone, lithium, or biotin use; pregnancy; other thyroid disease (such as goiter); or acute illness (such as infection)]. Additionally, we collected receipt and timing of the following TKIs associated with thyroid dysfunction: axitinib, cabozantinib, lenvantinib, pazopanib, sorafenib, sunitinib, and tivozanib.

We categorized thyroid function tests as normal or abnormal according to the institutional reference range: 0.4–4.5 mIU/L for TSH; 0.8–1.8 ng/dL for FT4. We defined baseline TSH and FT4 as the most recent laboratory result on or within 30 days prior to the first dose of ICI administered at the institution. Patients were considered to be on thyroid hormone replacement at baseline if they had an ongoing, active prescription on the day of ICI initiation. As done in earlier studies [18, 22, 23], we defined ICI-induced clinical thyroid dysfunction as any of the following: (1) initiation or any change in dose of levothyroxine treatment after ICI initiation; (2) a single substantial laboratory deviation from reference range indicating overt thyroid dysfunction (FT4 > 1.8 ng/dL or TSH > 10 mIU/L with FT4 < 0.8 ng/dL); or (3) ≥ 2 consecutive TSH values with unilateral deviation from reference range in patients with normal TSH at baseline. Any cases with the following clinical features were excluded specifically from the thyroid dysfunction analyses to minimize confounders: acute illness requiring hospitalization for any reason, pregnancy, existing thyroid disease (including thyroidectomy, goiter, thyroid nodules), or use of thyroid-disrupting medications including amiodarone, lithium, and biotin.

### Statistical analysis

Patients without baseline TSH values were excluded from the analysis. We compared demographic and clinical characteristics of patients with normal versus abnormal baseline TSH values using Fisher’s exact test and two-sample t-tests. We analyzed clinical outcomes according to baseline and post-ICI initiation lab values, clinical thyroid dysfunction, and treatment of thyroid dysfunction. We generated Kaplan–Meier curves to visualize OS differences between patient groups. Both the log-rank test and multivariate Cox proportional hazards model were used to test the association between OS and variables of interest. Overall *P* values in Cox proportional hazards model were determined using ANOVA. All survival analysis was performed using R package *survival* and *survminer*.

## Results

A total of 1897 patients were included, of which 1781 (94%) had a baseline TSH test (Table 1). Median age was 69 years, 62% were male, and 78% were white. The most common cancer types were lung, kidney, and melanoma. Sixteen patients (1%) had thyroid cancer. Additional demographic and clinical characteristics are shown in Table 1. Baseline TSH values were abnormal in 381 (21%), with values above reference range in 294 patients (16%) and below reference range in 87 patients (5%). As shown in Table 1, numerous clinical characteristics were associated with abnormal baseline TSH. These individuals were more likely to be female (*P* < 0.001), more likely to have kidney cancer (*P* < 0.001), and less likely to have melanoma (*P* < 0.001). We also analyzed the association between baseline TSH and other indicators of thyroid disease. Individuals with abnormal baseline TSH were more likely to have abnormal baseline FT4 (*P* < 0.001), although only 11% of those with abnormal baseline TSH had abnormal baseline FT4. Individuals with abnormal baseline TSH were also more likely to be receiving thyroid hormone replacement (44% vs 14%) (*P* < 0.001).

**Table 1:** Baseline demographics and clinical characteristics according to TSH status at baseline

Variable		Overall	Abnormal TSH at baseline	Normal TSH at baseline	*P*-value
Number of patients		1781	381	1400	
Age	Median (IQR)	69 (60, 75)	69 (60, 75)	69 (60, 75)	0.94
Gender, *N* (%)	Female	673 (38)	162 (43)	511 (37)	< 0.001
	Male	1108 (62)	219 (57)	889 (63)	
Race, *N* (%)	White	1392 (78)	304 (80)	1088 (78)	0.08
	Black	165 (9)	24 (6)	141 (10)	
	Asian	71 (4)	21 (6)	50 (4)	
	Other	153 (9)	32 (8)	121 (8)	
Ethnicity, *N* (%)	Hispanic	104 (6)	23 (6)	81 (6)	0.54
	Non-Hispanic	1602 (90)	346 (91)	1256 (90)	
	Unknown	75 (4)	12 (3)	63 (4)	
Cancer type, *N* (%)	Lung/bronchus	512 (29)	113 (30)	399 (29)	< 0.001
	Kidney	338 (19)	96 (26)	242 (17)	
	Skin/melanoma	238 (13)	33 (9)	205 (15)	
	Other	599 (34)	120 (31)	479 (34)	
	Unknown	94 (5)	19 (4)	75 (5)	
Abnormal TSH post baseline, *N* (%)	No (Normal)	751 (42)	61 (18)	690 (54)	< 0.001
	Yes (Abnormal)	863 (48)	286 (82)	577 (46)	
	Unavailable	167 (10)			
Abnormal FT4 at baseline, *N* (%)	No (Normal)	1547 (87)	331 (89)	1216 (97)	< 0.001
	Yes (Abnormal)	74 (4)	42 (11)	32 (3)	
	Unavailable	160 (9)			
Initiated Levothyroxine, *N* (%)	Never	1146 (64)	142 (37)	1004 (72)	< 0.001
	Prior to ICI	368 (21)	166 (44)	202 (14)	
	After to ICI	267 (15)	73 (19)	194 (14)	
ICI drug(s) at ICI initiation, *N* (%)	Anti-PD1/PDL1	1445 (81)	323 (84)	1122 (80)	0.12
	Anti-CTLA4	56 (3)	10 (3)	46 (3)	
	Anti-CTLA4 Plus anti-PD1/PDL1	280 (16)	48 (13)	232 (17)	

*CTLA4* cytotoxic T-lymphocyte antigen, *FT4 *free T4, *IC*— immune checkpoint inhibitor, *PD1* programmed cell death protein 1, *PDL1* programmed cell death-ligand 1, *TSH* thyroid stimulating hormone

**Fig. 1:**
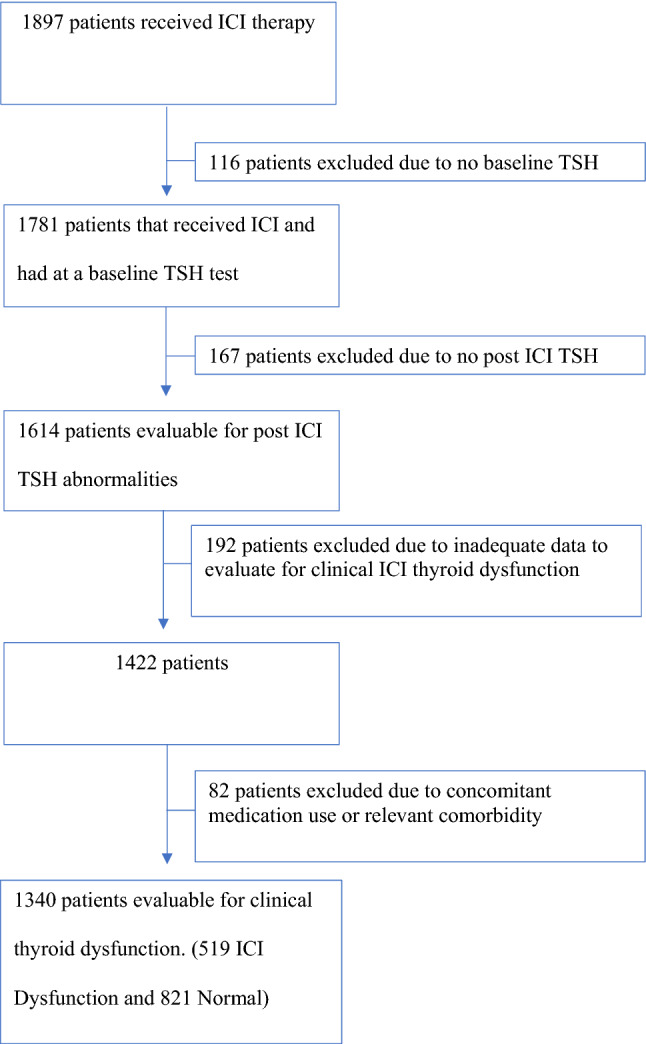
Consort diagram indicating inclusion and exclusion criteria for various analyses

In terms of clinical thyroid dysfunction, based on availability of laboratory and medication data to assess the three criteria described, 1422 patients were evaluable, of whom 82 (6%) were excluded due to concomitant medication use or relevant comorbidity as described in the methods (Fig. 1). Among the remaining 1340 patients, 519 (39%) were deemed to have clinical thyroid dysfunction after ICI initiation.

In addition to baseline TSH, post-ICI initiation TSH values were available for 1614 patients (85%), of whom 863 patients (53%) had one or more abnormal results (Table 2). Specifically, 492 (30%) had post-ICI initiation TSH values above the reference range, 204 (13%) had TSH values below the reference range, and 167 (10%) had TSH values both above and below the reference range. After ICI initiation, 267 patients (17%) started levothyroxine, while 23 patients (1%) received treatment for hyperthyroidism as follows: methimazole (*n* = 20), propylthiouracil (*n* = 2), both methimazole and propylthiouracil (*n* = 1). The occurrence of an abnormal TSH value post-ICI initiation was associated with gender, race, cancer type, and specific ICI therapy. Specifically, these individuals were more likely to be women, white, have kidney cancer, and be treated with combination anti-PD1 plus anti-CTLA4 treatment. As might be expected, patients with abnormal post-ICI initiation TSH values were more likely to have abnormal baseline thyroid parameters, including abnormal baseline TSH (33% vs 8%; *P* < 0.001), abnormal baseline FT4 (6% vs 2%; *P* < 0.001), and pre-ICI thyroid hormone replacement (31% vs 10%; *P* < 0.001).

**Table 2:** Baseline demographics and clinical characteristics according to TSH status post-ICI therapy

Variable		Overall	Abnormal TSH Post BL	Normal TSH Post BL	*P*-value
Number of patients		1614	863	751	
Age	Median (IQR)	69 (61, 76)	69 (61, 76)	69 (61, 76)	0.49
Gender, *N* (%)	Female	604 (37)	356 (41)	248 (33)	< 0.001
	Male	1010 (63)	507 (59)	503 (67)	
Race, *N* (%)	White	1267 (79)	697 (81)	570 (76)	0.02
	Black	154 (9)	66 (8)	88 (12)	
	Asian	59 (4)	27 (3)	32 (4)	
	Other	134 (8)	73 (8)	61 (8)	
Ethnicity, *N* (%)	Hispanic	98 (6)	57 (6)	41 (6)	0.51
	Non-Hispanic	1453 (90)	775(90)	678 (90)	
	Unknown	63 (4)	31 (4)	32 (4)	
Cancer Type, *N* (%)	Lung/bronchus	457 (28)	216 (25)	241 (32)	< 0.001
	Kidney	326 (20)	239 (28)	87 (12)	
	Skin/melanoma	224 (14)	121 (14)	103 (14)	
	Other	526 (33)	252 (29)	274 (36)	
	Unknown	81 (5)	35 (4)	46 (6)	
Abnormal TSH at baseline, *N* (%)	No (Normal)	1267 (79)	577 (67)	690 (92)	< 0.001
	Yes (Abnormal)	347 (21)	286 (33)	61 (8)	
Abnormal FT4 at baseline, *N* (%)	No (Normal)	1411 (87)	750 (94)	661 (98)	< 0.001
	Yes (Abnormal)	60 (4)	45 (6)	15 (2)	
	Unavailable	143 (9)			
Initiated Levothyroxine, *N* (%)	Never	1006 (62)	338 (39)	668 (89)	< 0.001
	Prior to ICI	341 (21)	268 (31)	73 (10)	
	After to ICI	267 (17)	257 (30)	10 (1)	
Thyroid dysfunction status, *N* (%)	ICI Dysfunction	519 (32)	487 (56)	32 (4)	< 0.001
	Normal	821 (51)	244 (29)	577 (77)	
	Excluded	274 (17)	132 (15)	142 (19)	
ICI drug(s) at ICI initiation, *N* (%)	anti-PD1/PDL1	1301 (81)	658 (76)	643 (86)	< 0.001
	anti-CTLA4	55 (3)	31 (4)	24 (3)	
	anti-CTLA4 plus anti-PD1/PDL1	258 (16)	174 (20)	84 (11)	

*CTLA4* cytotoxic T-lymphocyte antigen, *FT4* free T4, *ICI* immune checkpoint inhibitor, *PD1* programmed cell death protein 1, *PDL1* programmed cell death-ligand 1, *TSH* thyroid stimulating hormone

**Fig. 2:**
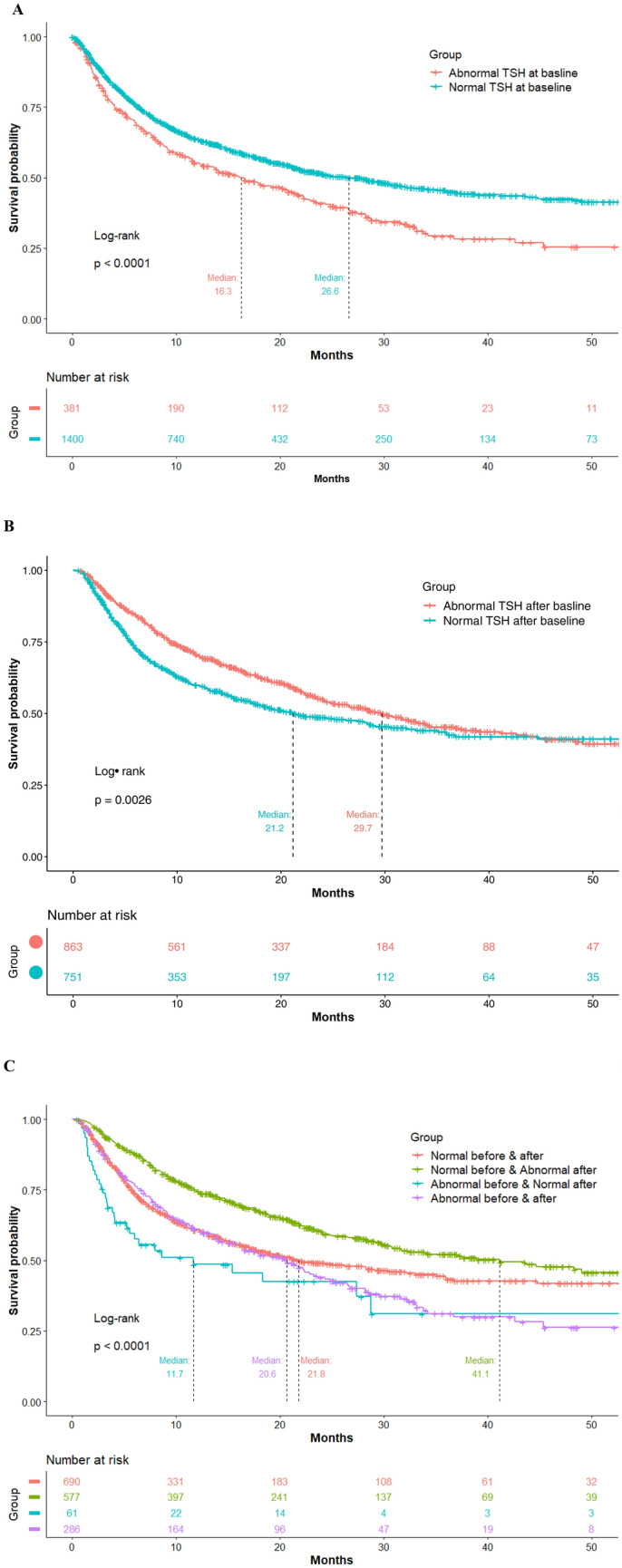
Overall survival according to **a** baseline TSH, **b** post-ICI initiation TSH, and **c** both baseline and post-ICI initiation TSH

Figure 2 displays overall survival according to baseline and post-ICI initiation TSH. Abnormal baseline TSH was associated with worse outcomes (median OS 16 months vs 27 months; *P* < 0.001) (Fig. 2a). Among these cases, both high (*n* = 294) and low (*n* = 87) baseline TSH values were associated with inferior OS (*P* < 0.001) (Supplemental Figure 1). In contrast, patients with abnormal TSH post-ICI initiation had better outcomes (Fig. 2b). When considering both baseline and post-ICI initiation TSH values, patients with normal baseline and abnormal post-ICI initiation TSH values had the best outcomes (median OS 41 months), while those with abnormal baseline and normal post-ICI initiation values had the worst outcomes (median OS 12 months) (*P* < 0.001) (Fig. 2c).

**Fig. 3:**
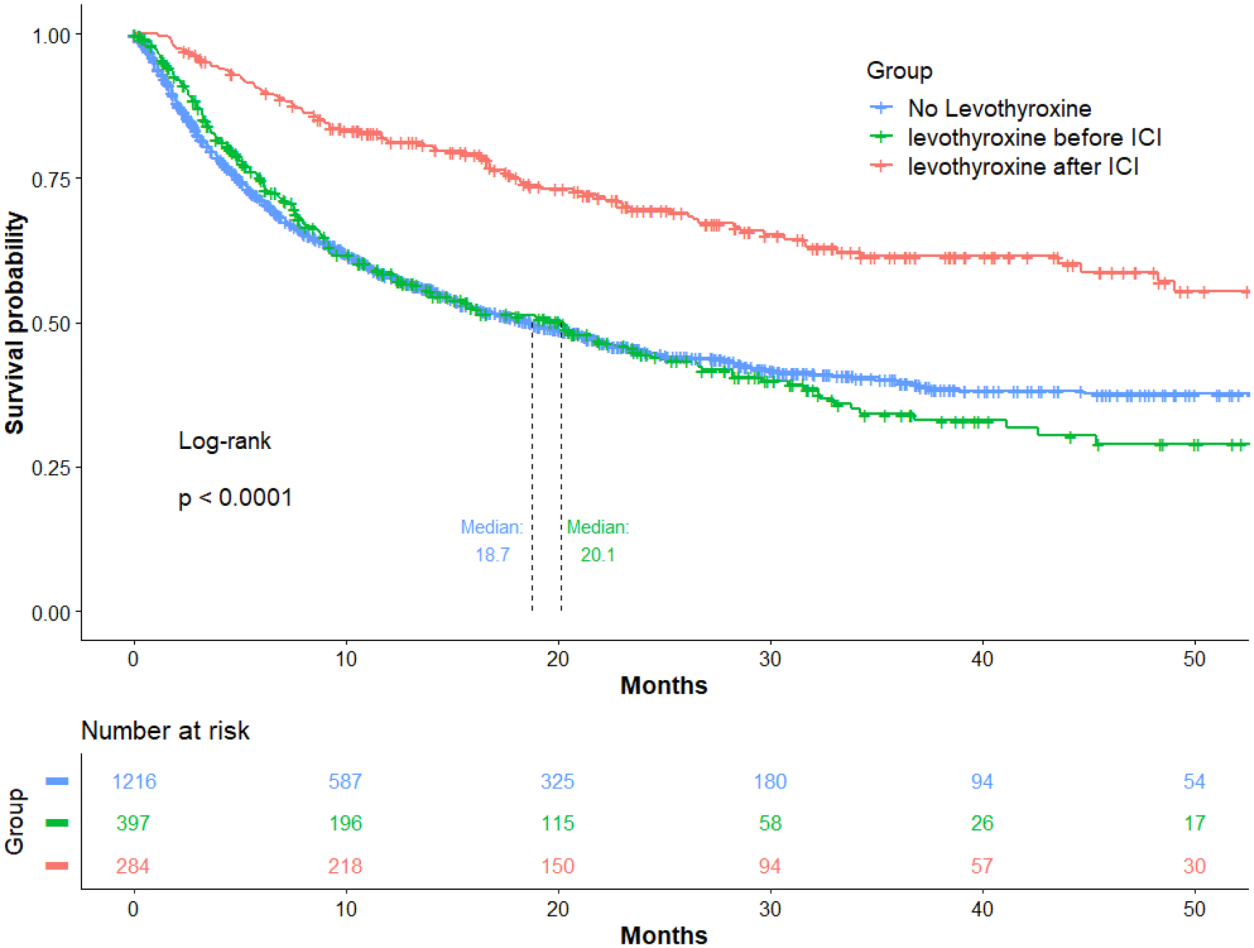
Overall survival according to levothyroxine use

**Fig. 4:**
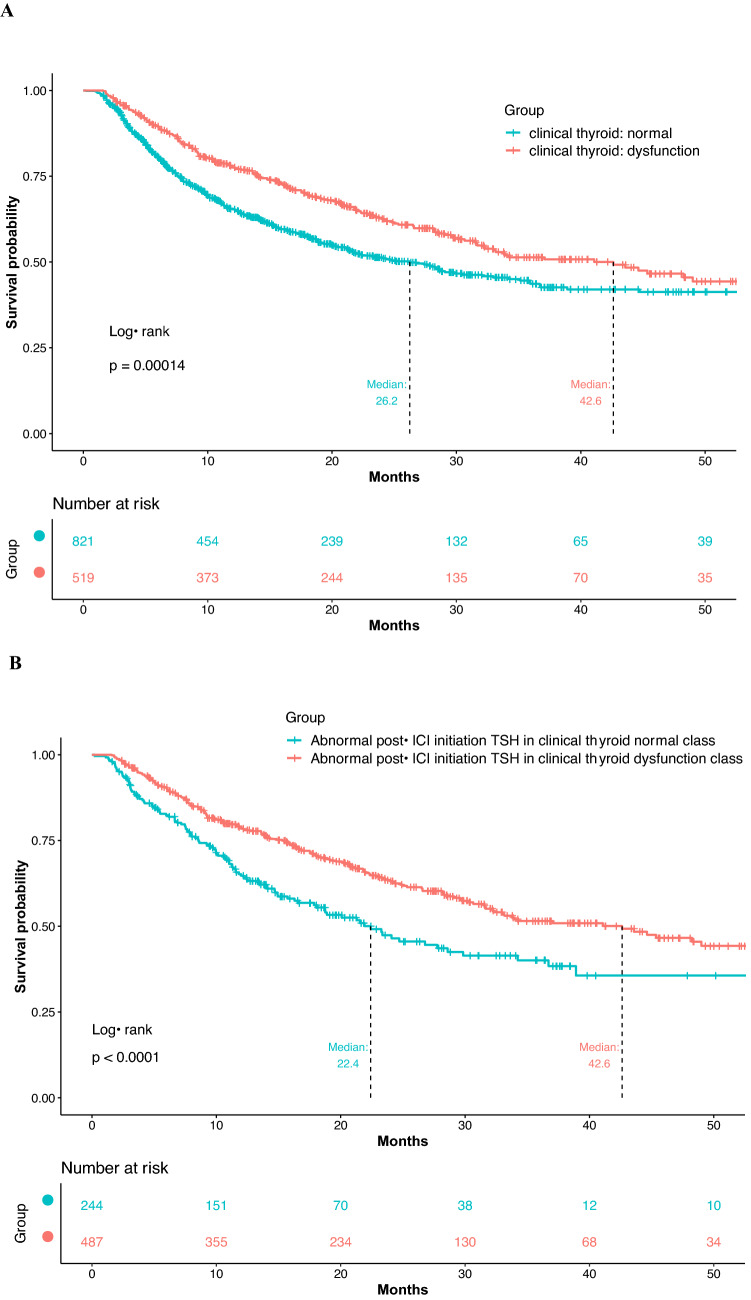
Overall survival according to **a** development of clinical thyroid dysfunction after ICI initiation, and **b** development of clinical thyroid dysfunction among those patients who developed abnormal TSH after ICI initiation

Patients initiated on levothyroxine after ICI initiation also had substantially improved OS (median not reached versus median 19 months) (*P* < 0.001) (Fig. 3). Conversely, those already on thyroid hormone replacement prior to ICI initiation had worse outcomes. Because abnormal TSH values occurred frequently in the study cohort and may have unclear clinical significance, we also examined outcomes according to (a) thyroid dysfunction criteria, and (b) receipt of thyroid hormone replacement. In general, these analyses yielded findings comparable to the TSH-based analyses. Development of ICI-induced thyroid dysfunction was associated with improved OS (median survival 43 months vs 26 months) (*P* < 0.001) (Fig. 4).

Given that renal cell carcinoma represents a distinct cancer type potentially treated with TKIs associated with thyroid toxicity, we performed sensitivity analyses of cohorts according to tumor type. Supplemental Figure 2 shows the same analyses performed in Figs. 2 and 3, with all RCC cases removed. Outcomes were similar to those observed in the overall cohort. We also performed the same analyses on RCC cases only (Supplemental Figure 3). Consistent with the overall cohort, abnormal TSH at baseline was significantly associated with worse OS, and initiation of levothyroxine post-ICI initiation was significantly associated with improved OS. However, analyses incorporating both baseline and post-ICI initiation TSH values were no longer significantly associated with outcomes.

To determine the effects of confounding therapies on our results, we also identified patients treated with TKIs associated with thyroid toxicity. Overall, 327 patients (17%) received at least one TKI as follows: pre-ICI initiation (*n* = 50, 2%), post-ICI initiation (*n* = 111, 6%), and both pre-and post-ICI initiation (*n* = 166, 9%). Receipt of specific TKIs was as follows: axitinib (*n* = 152, 8%), cabozantinib (*n* = 139, 7%), lenvatinib (*n* = 97, 5%), pazopanib (*n* = 77, 4%), sunitinib (*n* = 57, 3%), sorafenib (*n* = 22, 1%), tivozanib (*n* = 12, 1%). Some patients received more than one TKI. When these cases were removed from the overall cohort, we observed no meaningful difference in any outcomes (Supplemental Figure 4).

Table 3 displays the results of univariate and multivariate Cox regression analyses. In the multivariate Cox model, abnormal baseline TSH remained associated with worse OS (HR 1.62; 95% CI, 1.30–2.02; *P* < 0.001). Although abnormal post-ICI initiation TSH was not significantly associated with outcomes (HR 0.93; 95% CI, 0.74–1.12; *P* = 0.53), initiation of levothyroxine after ICI initiation remained associated with improved OS (HR 0.62; 95% CI, 0.44–0.88; *P* = 0.008). In the multivariate model, RCC diagnosis was associated with improved OS; however, TKI receipt was associated with worse OS. Given the clear overlap between RCC diagnosis and TKI receipt, it is difficult to interpret these individual results due to collinearity in the model.

**Table 3:** Overall survival Cox regression analyses

Covariate	Univariate analysis			Multivariate analysis		
	HR (95% CI)	*P* value	Overall *P* value	HR (95% CI)	*P* value	Overall *P* value
Age (continuous variable)	1 (0.99, 1.01)	0.133		1 (0.99, 1.01)	0.44	
*Gender*						
Male	0.94(0.79, 1.12)	0.53		1.05 (0.87, 1.25)	0.63	
Female	Reference					
*Race*						
White	1.4 (0.98, 2.0)	0.06	0.05	1.34 (0.89, 2.01)	0.15	0.05
Black	1.79 (1.16, 2.75)	0.008		1.38 (0.86, 2.21)	0.19	
Asian	1.11 (0.61, 2.02)	0.74		0.83 (0.44, 1.56)	0.56	
Unknown	Reference					
*Ethnicity*						
Hispanic	0.98 (0.48, 2.00)	0.95	0.03	0.92 (0.44, 1.93)	0.82	0.11
Non-Hispanic	1.57 (0.87, 2.86)	0.14		1.34 (0.69, 2.58)	0.38	
Unknown	Reference					
*Cancer type*						
Lung	1.03(0.65, 1.65)	0.89	< 0.001	1.17 (0.72, 1.88)	0.53	< 0.001
Melanoma	0.45 (0.27, 0.75)	< 0.001		0.52 (0.30, 0.89)	0.02	
Kidney	0.65 (0.40, 1.05)	0.08		0.42 (0.25, 0.71)	0.001	
Other	1.16 (0.73, 1.84)	0.52		1.25 (0.78, 1.99)	0.36	
Unknown	Reference					
*ICI drug*						
Anti-CTLA4	0.53 (0.33, 0.83)	0.001	< 0.001	1.04 (0.62, 1.72)	0.89	0.79
Anti-CTLA4 plus Anti-PD1/PDL1	0.66 (0.52, 0.88)	0.006		1.18 (0.89, 1.58)	0.25	
Anti-PD1/PDL1	Reference					
*Baseline TSH*						
Abnormal	1.49 (1.24, 1.81)	< 0.001		1.62 (1.30, 2.02)	< 0.001	
Normal	Reference					
*Post baseline TSH*						
Abnormal	0.84 (0.71, 0.99)	0.03		0.93 (0.74, 1.12)	0.53	
Normal	Reference					
*Clinical thyroid status*						
Abnormal	0.72 (0.60, 0.85)	< 0.001		0.88 (0.66, 1.17)	0.38	
Normal	Reference					
*Levothyroxine use*						
Initiated prior to ICI	1.19 (0.97, 1.46)	0.08	< 0.001	1.13 (0.86, 1.48)	0.37	< 0.001
Initiated after ICI	0.51 (0.39. 0.66)	< 0.001		0.62 (0.44, 0.88)	0.008	
Never	Reference					
*TKI*^a^						
None	0.97(0.75, 1.25)	0.79	0.2	0.52 (0.35, 0.75)	< 0.001	< 0.001
Post-ICI initiation	0.79(0.53, 1.19)	0.26		0.72 (0.46, 1.11)	0.14	
Pre-ICI initiation	1.49(0.88, 2.51)	0.13		1.13 (0.78, 2.25)	0.30	
Pre- and post-ICI initiation	Reference					

*CTLA4* cytotoxic T-lymphocyte antigen, *FT4* free T4, *ICI* immune checkpoint inhibitor, *PD1* programmed cell death protein 1, *PDL1* programmed cell death-ligand 1, *TKI* tyrosine kinase inhibitor, *TSH* thyroid stimulating hormone

^a^Receipt of a TKI associated with possible thyroid toxicity (axitinib, cabozantinib, lenvantinib, pazopanib, sorafenib, sunitinib, or tivozanib)

## Discussion

Thyroid dysfunction represents one of the most common adverse effects of checkpoint inhibitor therapy. Furthermore, because the diagnosis relies upon discrete laboratory test results and there is no other likely cause for acute derangements in these parameters, immune-related thyroid disorders appear to be easier to diagnose and characterize than most other irAE [24]. Thyroid dysfunction is also readily managed, rarely requiring any intervention beyond thyroid hormone replacement, although this treatment may be lifelong [1]. In the present study, we capitalized on the widespread practice of monitoring thyroid function tests prior to and during cancer immunotherapy to determine correlates and prognostic effects of thyroid dysfunction in cancer patients treated with ICI.

Notably, over 20% of our cohort had abnormal TSH at baseline, although fewer than half of these individuals had abnormal FT4. Arguably a better indicator of pre-existing clinical thyroid disease, over 20% of patients were receiving thyroid hormone replacement (levothyroxine) prior to immunotherapy, with slightly more than half of these individuals having a normal TSH at time of ICI initiation. These rates are substantially higher than observed in the general population, in which prevalence of hypothyroidism ranges 2–5% [25, 26]. One contributing factor may be the advanced age of individuals with cancer, as hypothyroidism is more common in those age > 65 years [27]. Additionally, thyroid disease occurs more frequently among white individuals [28], who made up more almost 80% of our study population. Additional patient intrinsic factors that may affect irAE risk include host genomics and epigenomics, host immunity, and the tumor microenvironment [29].

Within our study cohort, women were more likely to have abnormal baseline TSH, consistent with the broader epidemiology of thyroid disease [12, 27]. Interestingly, baseline TSH abnormalities were also more common in patients with kidney cancer, which could reflect prior treatment with certain TKIs associated with thyroid dysfunction. For instance, hypothyroidism occurs in more than 25% of patients treated with sunitinib or with lenvatinib plus everolimus [30]. This becomes a particularly relevant consideration when combining these agents with checkpoint inhibitors, as the risk of hypothyroidism is almost 50% in patients treated with lenvatinib plus pembrolizumab [30]. Additionally, approximately half of our cohort receiving anti-PD-1 or anti-PD-L1 therapy developed abnormal TSH after treatment. This rate is higher than has been reported in ICI clinical trials, but is consistent with recently published real-world studies [31], which may reflect different thresholds for reporting.

Although RCC may be treated with certain TKIs affecting thyroid function, neither RCC diagnoses nor TKI administration appear to account for our findings. Sensitivity analyses excluding RCC cases and excluding patients receiving these TKIs showed similar results as the overall cohort. While some of our significant observations were not replicated in the RCC-only cohort analysis, it is not clear whether these distinctions represent true clinical or biologic differences, or loss of statistical power due to smaller sample size.

Perhaps the most striking finding of this study, baseline abnormal TSH was associated with clinically and statistically significantly worse outcomes, an observation supported by the trend toward worse outcomes in those receiving levothyroxine before ICI initiation. In general populations, studies of long-term prognostic effects of thyroid disorders have been largely limited to subclinical hypothyroidism, as overt hypothyroidism and hyperthyroidism are usually treated. While subclinical hypothyroidism (particularly for TSH > 10 mIU/L) is associated with an increased risk of coronary heart disease, it is not associated with increased total mortality [32]. In the elderly, some studies have identified detrimental effects of subclinical hypothyroidism on survival [33], while others suggest that individuals with high TSH may have prolonged life spans [34] and that treatment of subclinical hypothyroidism may increase mortality [35]. Little is known about the impact of thyroid status on cancer-related outcomes. A single population study from Taiwan found that patients with subclinical hypothyroidism were more likely to have bone, skin, or breast cancer, and that subclinical hypothyroidism was associated with increased risk of cancer death in the elderly, women, and heavy smokers [36]. While abnormal TSH could hypothetically reflect non-thyroidal illness [37], this seems unlikely in our study cohort because patients with severe acute illness such as sepsis would generally not be considered suitable to initiate systemic anti-cancer therapy.

Why might baseline TSH abnormalities be associated with inferior clinical outcomes in patients receiving ICI? In iodine-sufficient areas, chronic autoimmune thyroiditis is the most common cause of hypothyroidism, which has a clinical association with other autoimmune diseases including celiac disease and type 1 diabetes [11]. Furthermore, it is estimated that TSH concentrations are 65% heritable, with autoimmunity-related genes (e.g., HLA class I region, *PTPN22*, *SH2B3*, and *VAV3*) among the most consistently implicated loci [38]. The influence of prior autoimmune disease on checkpoint inhibitor efficacy remains unclear. While one might theorize that patients with pre-existing autoimmunity may be more likely to benefit from these treatments, an alternative hypothesis is that such patients have defective anti-tumor immunity, as malignancy developed despite a heightened immune state. Existing clinical data may be difficult to interpret, as patients with other pre-existing autoimmune conditions may be receiving systemic immunosuppression before and/or during ICI treatment. These individuals also appear to be more likely to discontinue immunotherapy, either due to irAE or flare of the underlying autoimmune condition [39]. However, along with type 1 diabetes and Addison disease, thyroid dysfunction is managed distinctly from most other autoimmune diseases in that patients do not receive immunosuppression. Therefore, if they develop cancer, they do so despite a heightened immune state. Perhaps ICI is less likely to benefit such individuals. Conversely, as noted in earlier reports [18, 19], we found that individuals who developed ICI-induced thyroid dysfunction had improved outcomes, presumably because they experienced greater immune activation.

As reported previously [40], abnormal baseline TSH was associated with post-ICI initiation thyroid abnormalities. Comparable to rates of thyroid laboratory aberrations and clinical thyroid disease in the general population, only about half of patients who developed post-ICI initiation TSH abnormalities were eventually diagnosed with clinical thyroid dysfunction. Importantly, this subset of individuals had clearly improved outcomes compared to patients with treatment-emergent TSH abnormalities not meeting the threshold for clinical thyroid dysfunction. If ICI-induced thyroid disorders, similar to other irAE, represent heightened immune response to checkpoint inhibitors, it seems possible that isolated, mild laboratory abnormalities could also reflect other mechanisms, including random variation, medication use, timing of blood collection [11], seasonal variation [41], or non-thyroidal illness. In such individuals, then, the TSH abnormality would not reflect immune activation.

Limitations of this study include a single-center setting, the retrospective nature of data collection, and the lack of certain clinical variables such as response rate, progression-free survival, and other irAE. Furthermore, because TSH is not routinely assessed before or during most other types of cancer therapies (including surgery, radiation therapy, chemotherapy, and most molecularly targeted agents), we are not able to determine the extent to which our findings are specific to immunotherapy. We are unable to discern the etiology of baseline thyroid function test abnormalities. We do not have available clinical data on comorbidity burden, specific ICI dosing strategy, number of therapeutic lines, or PDL1 expression. For thyroid replacement, we limited data collection to levothyroxine because this medication represents standard practice and only a small proportion of patients typically use other thyroid replacement modalities such as desiccated animal thyroid extract [42]. We also recognize that dose changes of previously prescribed levothyroxine post-ICI initiation may not necessarily reflect ICI effects. Additionally, we recognize the possibility that some patients may have received ICI at another institution previously, in which case the pre-ICI lab tests would not represent a true baseline. However, because sequential ICI regimens are rarely used clinically, this would likely be a rare occurrence. Study strengths include the large sample size, the availability of pre- and post-ICI thyroid function tests, and the distinction between clinical thyroid dysfunction and laboratory-limited abnormalities.

In conclusion, pre-existing and treatment-emergent thyroid dysfunction appear to have divergent prognostic effects in patients treated with ICI. As seen with other irAE, individuals who develop ICI-induced thyroid disease have improved outcomes. This effect appears limited to those patients who experience clinical thyroid disease syndrome rather than isolated laboratory aberrations. Importantly, pre-existing thyroid dysfunction—whether defined as requiring treatment or having an abnormal baseline TSH—is associated with worse survival, even after controlling for multiple clinical, tumor-, and treatment-related variables. Given the prevalence of thyroid disease in cancer populations, further research into potential explanations for this observation are warranted.

## Supplementary Information

**Supplemental Figure 1.** Overall survival according to baseline high versus low TSH.

**Supplemental Figure 2.** Sensitivity analyses excluding renal cell carcinoma cases: (A) overall survival according to baseline TSH, (B) overall survival according to post-ICI initiation TSH, (C) overall survival according to both baseline and post-ICI initiation TSH, and (D) overall survival according to levothyroxine use.

**Supplemental Figure 3.** Sensitivity analyses including only renal cell carcinoma cases: (A) overall survival according to baseline TSH, (B) overall survival according to post-ICI initiation TSH, (C) overall survival according to both baseline and post-ICI initiation TSH, and (D) overall survival according to levothyroxine use.

**Supplemental Figure 4.** Sensitivity analyses excluding cases receiving selected tyrosine kinase inhibitors: (A) overall survival according to baseline TSH, (B) overall survival according to post-ICI initiation TSH, (C) overall survival according to both baseline and post-ICI initiation TSH, and (D) overall survival according to levothyroxine use.

Below is the link to the electronic supplementary material.

Supplementary file1 (DOCX 48 kb)

Supplementary file2 (DOCX 134 kb)

Supplementary file3 (DOCX 75 kb)

Supplementary file4 (DOCX 137 kb)

## Data Availability

De-identified data will be made available upon reasonable request to corresponding author.
