# Correction to: Divergent prognostic effects of pre-existing and treatment-emergent thyroid dysfunction in patients treated with immune checkpoint inhibitors

**DOI:** 10.1007/s00262-022-03218-0

**Published:** 2022-05-21

**Authors:** Mitchell S. von Itzstein, Amrit S. Gonugunta, Yiqing Wang, Thomas Sheffield, Rong Lu, Sadia Ali, Farjana J. Fattah, Donglu Xie, Jennifer Cai, Yang Xie, David E. Gerber

**Affiliations:** 1 Department of Internal Medicine, Division of Hematology and Oncology, Harold C. Simmons Comprehensive Cancer Center, University of Texas, Southwestern Medical Center, 5323 Harry Hines Blvd., Mail Code 8852, Dallas, TX 75390-8852 USA; 2 Harold C. Simmons Comprehensive Cancer Center, University of Texas, Southwestern Medical Center, Dallas, TX 75390 USA; 3 School of Medicine, University of Texas, Southwestern Medical Center, Dallas, TX 75390 USA; 4 Department of Population and Data Sciences, University of Texas, Southwestern Medical Center, Dallas, TX 75390 USA; 5 Department of Internal Medicine, Division of Endocrinology, University of Texas, Southwestern Medical Center, Dallas, TX 75390 USA; 6 Academic Information Systems, University of Texas, Southwestern Medical Center, Dallas, TX 75390 USA; 7 Present Address: Stanford University, Palo Alto, CA USA

## Correction to: Cancer Immunology, Immunotherapy 10.1007/s00262-022-03151-2

The article Divergent prognostic effects of pre‑existing and treatment‑emergent thyroid dysfunction in patients treated with immune checkpoint inhibitors, written by Mitchell S. von Itzstein, Amrit S. Gonugunta, Yiqing Wang, Thomas Sheffield, Rong Lu, Sadia Ali, Farjana J. Fattah, Donglu Xie, Jennifer Cai, Yang Xie and David E. Gerber, was originally published electronically on the publisher’s internet portal on 24 January 2022 without open access. With the author(s)’ decision to opt for Open Choice the copyright of the article changed on 26 April 2022 to © The Author(s) 2021 and this article is licensed under a Creative Commons Attribution 4.0 International License, which permits use, sharing, adaptation, distribution and reproduction in any medium or format, as long as you give appropriate credit to the original author(s) and the source, provide a link to the Creative Commons licence, and indicate if changes were made. The images or other third party material in this article are included in the article's Creative Commons licence, unless indicated otherwise in a credit line to the material. If material is not included in the article's Creative Commons licence and your intended use is not permitted by statutory regulation or exceeds the permitted use, you will need to obtain permission directly from the copyright holder. To view a copy of this licence, visit http://creativecommons.org/licenses/by/4.0/

The original article has been corrected.

