# Supplementary material for: Divergent prognostic effects of pre-existing and treatment-emergent thyroid dysfunction in patients treated with immune checkpoint inhibitors
Source: Cancer Immunol Immunother. 2022 Jan 24;71(9):2169–81. doi: 10.1007/s00262-022-03151-2 (PMC9308834; doi:10.1007/s00262-022-03151-2)
Supplement: Supplementary file 2 — Supplementary file2 (DOCX 134 kb) [file 262_2022_3151_MOESM2_ESM.docx]

**Supplemental Figure 2**

**A**

**B**

**C**

**D**
